# Editorial: Current and Emerging Trends in Human Identification and Molecular Anthropology

**DOI:** 10.3389/fgene.2021.708222

**Published:** 2021-06-23

**Authors:** Cemal Gurkan, Ozlem Bulbul, Kenneth K. Kidd

**Affiliations:** ^1^Turkish Cypriot DNA Laboratory, Committee on Missing Persons in Cyprus Turkish Cypriot Member's Office, Nicosia, Turkey; ^2^Dr. Fazil Küçük Faculty of Medicine, Eastern Mediterranean University, Famagusta, Turkey; ^3^Institute of Forensic Science, Istanbul University-Cerrahpasa, Istanbul, Turkey; ^4^Department of Genetics, Yale University School of Medicine, New Haven, CT, United States

**Keywords:** ancestry informative markers, forensic DNA phenotyping, age prediction, microhaplotypes, genotyping

Recent developments in the DNA analysis technologies such as Massively Parallel Sequencing (MPS) have found prolific use in forensic applications. MPS not only allows the identification of a given individual using traditional forensic genetic markers but also the prediction of his/her age, appearance, and ancestry (Børsting and Morling, 2015). This can be very useful when conventional identification attempts using the comparative DNA profiling systems [e.g., short tandem repeats (STRs)] reach a dead end due to the absence of requisite DNA reference samples and/or potential matches in the respective databases. DNA-based estimation of ancestry and physical characteristics, now collectively described as forensic DNA phenotyping (FDP), has also led to the emergence of the concept of “biological witness” that can potentially provide investigative leads in such cases (Kayser, 2015). For instance, the prediction of the eye and hair color of King Richard III of England (1452–1485) and World Word II victims were all made possible with FDP (King et al., 2014; Chaitanya et al., 2017). A growing number of allele frequency data for diverse reference populations has facilitated increasingly better estimates of individual biogeographic ancestry (Pakstis et al., 2015, 2019; Bulbul et al., 2018). In other words, FDP may help us better understand the recent or distant past *via* the “reconstruction” of otherwise unidentifiable human remains. Many facets of human identification in a forensic context overlap with various aspects of human population genetics and molecular anthropology. Accordingly, in this Research Topic, we sought manuscripts that would provide a snapshot of the current and emerging trends in human identification or molecular anthropology, especially at the interface of the two fields (Figure 1).

**Figure 1:**
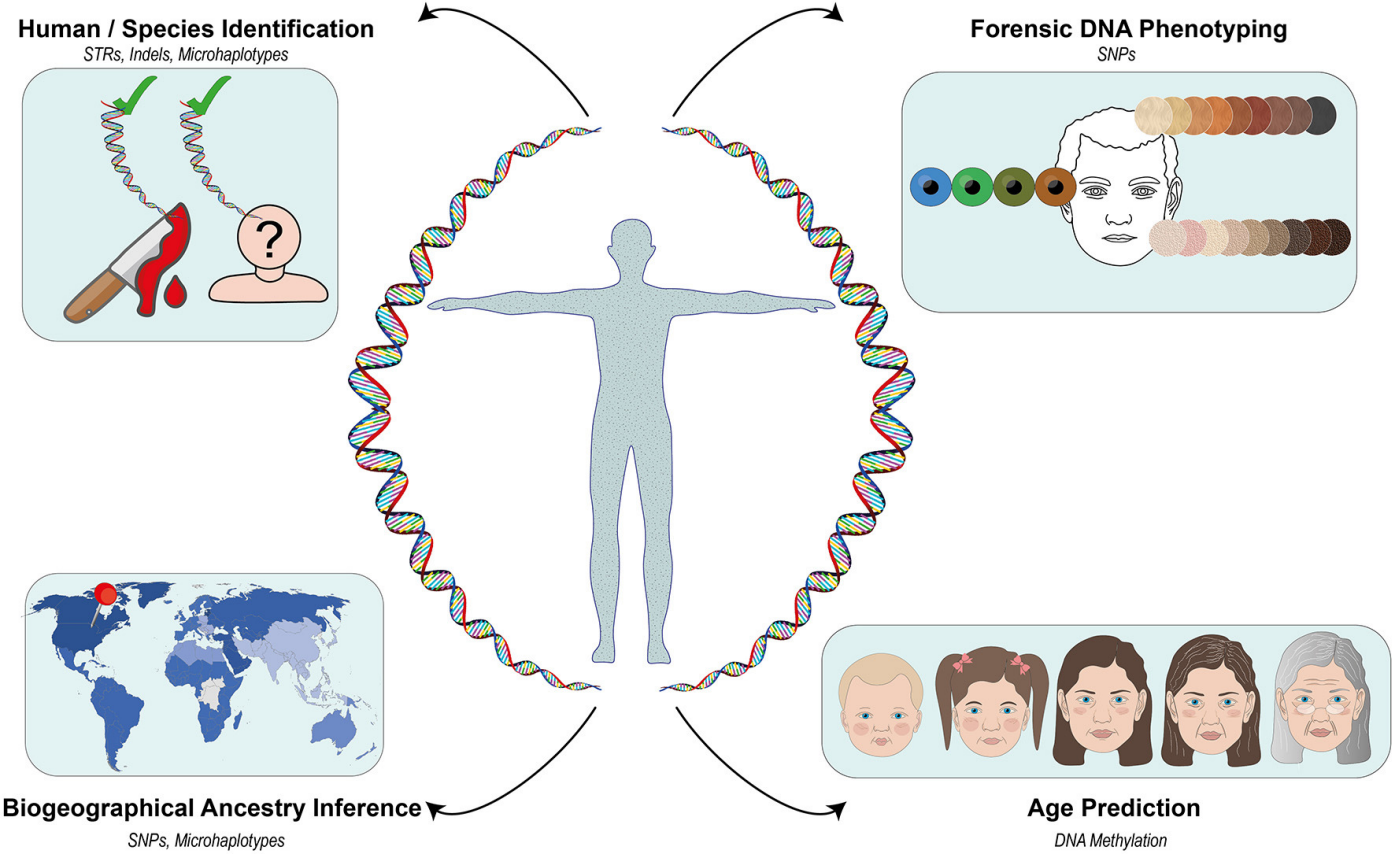
An overview of the genetic markers covered in the Current and Emerging Trends in Human Identification and Molecular Anthropology Research Topic.

A significant new trend in human identification and molecular anthropology is the emergence of a new genetic marker called *microhaplotypes*. Since the introduction of the concept less than a decade ago (Kidd et al., 2013), these very short segments (<300 bp) of the genome with multiple variants such that the loci are multiallelic have been getting increasing attention (Oldoni et al., 2019). Use of microhaplotypes is exemplified by de la Puente et al. who present a validation of a panel of 113 loci showing their power for biogeographic ancestry inference as well as familial relationship detection. Furthermore, Qu et al. have broadened the concept of the microhaplotype to include multiple small insertions/deletions allowing the markers to be studied using a conventional method like capillary electrophoresis (CE). Accordingly, these new microhaplotype loci may provide a useful supplement to standard CE-based STR typing using the same equipment. The growing number of published microhaplotype loci and the accumulating population data on them has been summarized in a new database called MicroHapDB by Standage and Mitchell. This extensible database will certainly be an important resource as the trend for more microhaplotype studies continues.

Another emerging trend is the gradual introduction of FDP in more routine casework investigations, and to this end, Atwood et al., report the selection process for the provision of FDP services to be used by the Australian law enforcement agencies. Here, a comparison of FDP services offered by six different providers was carried out that led to the successful selection of a provider for the prediction of biogeographical ancestry, hair, and eye color. A parallel trend in forensic research, which can also be regarded complementary to FDP, is the use of molecular techniques to determine the age of the person who left a DNA sample (Naue et al., 2018). Freire-Aradas et al. present a methodological comparison of the current DNA-methylation based forensic age prediction models using four different technologies available, and report largely comparable age predictions results. In fact, DNA methylation-based investigations are part of the growing field of forensic epigenomics whereby it has already been shown to distinguish homo-zygotic twins and the source tissue of a given sample, but also in the near future, even hold the potential for the prediction of lifestyle and environmental exposures of unknown perpetrators (Vidaki and Kayser, 2017).

Of course, studies on more traditional markers such STRs is still ongoing with several new applications. Cui et al. have taken STRs beyond humans to identify loci that can be used to identify different species of interest in a given sample. Their panel can identify DNA samples from 10 different species in addition to human using pairs of species-specific STR loci, and as such it may not only find use in forensic species identification, but also in the detection of meat fraud and adulteration. Gomes et al. focus on the unique inheritance and population genetic characteristics of markers located on the X chromosome. While noting an unexpected decrease in the number of new forensic investigations in the literature over the last two decades using X chromosome markers, nevertheless, the authors show the utility of these markers, primarily STRs, that so far have been identified. On a different note, Wyner et al. question the validity of the common assumption that the standard forensic STR loci are not associated with any phenotypes. In the light of increasing amount of compelling evidence toward the associations between forensic STR loci and certain phenotypes, authors point out to the presence of numerous legal and ethical implications associated with the already accumulated and expanding data using these markers, and suggest follow-ups and appropriate counter measures to minimize any misuse of such additional information.

SNPs continue to be investigated for their ability to infer ancestry of individuals and genetic affinities between populations. Pereira et al. have studied one admixed population in Brazil and shown dependencies in assessing admixture in ancestry of individuals on the numbers of markers used and the criteria used to identify the markers. Liu et al. have studied many populations in East Asia to assess the ancestry of Tibetans and their relationships to other Asian populations.

## Author Contributions

CG, OB, and KK co-edited the Research Topic and co-wrote, co-edited, and co-approved the final version of the Editorial for publication. All authors contributed to the article and approved the submitted version.

## Conflict of Interest

The authors declare that the research was conducted in the absence of any commercial or financial relationships that could be construed as a potential conflict of interest.
